# Dysregulation of the *TCF4* Isoform in Corneal Endothelial Cells of Patients With Fuchs Endothelial Corneal Dystrophy

**DOI:** 10.1167/iovs.65.6.27

**Published:** 2024-06-17

**Authors:** Tetsuro Honda, Tatsuya Nakagawa, Taichi Yuasa, Yuichi Tokuda, Masakazu Nakano, Kei Tashiro, Theofilos Tourtas, Ursula Schlötzer-Schrehardt, Friedrich Kruse, Koji Yamamoto, Noriko Koizumi, Naoki Okumura

**Affiliations:** 1 Department of Biomedical Engineering, Faculty of Life and Medical Sciences, Doshisha University, Kyotanabe, Japan; 2 Department of Genomic Medical Sciences, Kyoto Prefectural University of Medicine, Kyoto, Japan; 3 Department of Ophthalmology, University of Erlangen-Nürnberg, Erlangen, Germany

**Keywords:** fuchs endothelial corneal dystrophy, RNA-Seq, isoform, differential exon usage, TCF4

## Abstract

**Purpose:**

This study evaluated the dysregulation of *TCF4* isoforms and differential exon usage (DEU) in corneal endothelial cells (CECs) of Fuchs endothelial corneal dystrophy (FECD) with or without trinucleotide repeat (TNR) expansion in the intron region of the *TCF4* gene.

**Methods:**

Three RNA-Seq datasets of CECs (our own and two other previously published datasets) derived from non-FECD control and FECD subjects were analyzed to identify *TCF4* isoforms and DEU events dysregulated in FECD by comparing control subjects to those with FECD with TNR expansion and FECD without TNR expansion.

**Results:**

Our RNA-Seq data demonstrated upregulation of three *TCF4* isoforms and downregulation of two isoforms in FECD without TNR expansion compared to the controls. In FECD with TNR expansion, one isoform was upregulated and one isoform was downregulated compared to the control. Additional analysis using two other datasets identified that the *TCF4-277* isoform was upregulated in common in all three datasets in FECD with TNR expansion, whereas no isoform was dysregulated in FECD without TNR expansion. DEU analysis showed that one exon (E174) upstream of the TNR, which only encompassed *TCF4-277*, was upregulated in common in all three datasets, whereas eight exons downstream of the TNR were downregulated in common in all three datasets in FECD with TNR expansion.

**Conclusions:**

This study identified *TCF4-277* as a dysregulated isoform in FECD with TNR expansion, suggesting a potential contribution of *TCF4-277* to FECD pathophysiology.

Fuchs endothelial corneal dystrophy (FECD) is an inherited eye disease that affects the corneal endothelium of bilateral eyes.^1^^–^^3^ The formation of excrescences called guttae between the corneal endothelial cells (CECs) and the basement membrane (Descemet's membrane) is a clinical FECD hallmark that reduces contrast sensitivity and increases glare.^4^^,^^5^ The CECs maintain corneal transparency by barrier and pump function; therefore the damage induced by FECD results in severe vision loss because of corneal edema when the disease progresses to the late stage. Transplantation of donor corneas has been the most common treatment for FECD,^6^ but the multitude of problems associated with corneal transplantation, including worldwide donor shortage, surgical invasion, and donor cornea rejection, have led researchers to search for and develop alternative therapies.^7^^–^^11^

FECD is accepted as the most common genetic corneal disease; however, its genetic cause remains unelucidated at present, except for a few potential genetic variants for minor populations.^2^^,^^12^^–^^14^ This lack of knowledge of the causative gene for the major population with FECD has hampered the elucidation of the pathophysiology of this disease. However, in 2012, Wieben and colleagues^15^ reported that 79% of the patients with FECD in their study harbored an expansion of the CTG trinucleotide repeat (TNR) ≥ 50, whereas only 3% of their non-FECD control subjects harbored the TNR expansion. This landmark discovery of a common genomic variant shed light on the pathology of FECD, and subsequent research based on this discovery proposed the following hypothetical mechanisms underlying FECD: (1) dysregulated expression of *TCF4* transcripts; (2) toxicity of the TNR primary RNA transcripts; (3) repeat-associated non-AUG translation; and (4) TNR length instability.^16^

Our previous quantitative PCR (qPCR) study showed an upregulation of the *TCF4* mRNA level in the CECs of patients with FECD compared to non-FECD subjects.^17^ However, other studies reported inconsistent results for the expression levels of *TCF4* mRNA in CECs of patients with FECD.^18^^–^^20^ This discrepancy was considered to probably reflect the limitations of qPCR, because *TCF4* has at least 93 isoforms with variable expression levels.^21^^–^^23^ Indeed, subsequent RNA sequencing (RNA-Seq) revealed that the expression levels of the many *TCF4* isoforms were increased or decreased simultaneously in the CECs of patients with FECD with TNR expansion.^22^ These RNA-Seq findings led to the proposal of a potential contribution of a TNR to the dysregulated expression of the *TCF4* isoforms.^22^

In the present study, we conducted a further investigation of the dysregulated *TCF4* isoforms using three RNA-Seq datasets, including one of ours,^24^^,^^25^ that are currently available in the repository. We identified *TCF4* isoforms commonly dysregulated in FECD in all three available datasets. We also explored differential exon usage (DEU) in *TCF4*.

## Material and Methods

### Ethics Statement

Human samples were handled following guidelines based on the ethical principles of the Declaration of Helsinki. This study was performed according to a protocol approved by the ethical review committee of the Doshisha University Ethics Committee for Scientific Research Involving Human Subjects (Approval no. 20009), the Institutional Review Board of Kyoto Prefectural University of Medicine (Approval no. ERB-G-73), and the Friedrich-Alexander Universität Erlangen-Nürnberg (Approval no. 140_20 B). Informed consent to obtain peripheral blood samples and Descemet's membranes, including CECs, was acquired at the Friedrich-Alexander Universität Erlangen-Nürnberg.

### Acquisition of RNA-Seq Data

We utilized our previously reported RNA-Seq dataset for CECs derived from non-FECD control^24^ and FECD subjects^25^ (hereinafter defined as the “Nakagawa 2023” dataset). The raw fastq files for CECs derived from non-FECD and FECD subjects were obtained from the DNA Data Bank of Japan Sequence Read Archive under accession ID: DRP006678^24^ and DRA015078.^25^ Sample information is provided in Supplementary Table S1.

We also used two additional RNA-Seq datasets for CECs from non-FECD control subjects and patients with FECD for further analyses. One dataset (hereinafter defined as the “Nikitina 2019” dataset)^26^ was downloaded from an NCBI database (BioProject accession number PRJNA524323). Note that the sample of “Dfu_212” (Sequence Read Archive (SRA) ID: SRX5431504) from the Nikitina 2019 dataset, which harbors 44 repeat expansions in *TCF4*, was originally classified as an expansion group (Supplementary Table S2). Another dataset (hereinafter defined as the “Chu 2020” dataset)^27^ was also downloaded from an NCBI database (BioProject accession number PRJNA597343). Note that the “Control_8” sample (SRA ID: SRX5431504) in the Chu 2020 dataset was excluded from our analysis because of its low average spot length (Supplementary Table S3).

### RNA-Seq Data Processing

Low-quality reads were eliminated from the raw fastq files in each dataset using the fastp program (v0.20.0),^28^ and the raw fastq files were subjected to quality control using the FastQC program (v0.11.9; Babraham Bioinformatics). The reads were then aligned to the human reference genome (Homo_sapiens.GRCh38.104) by STAR (v2.7.10a).^29^ The Ensembl annotation file (GRCh38.104.gtf) was applied as the reference annotations for all genes. The read counts for each isoform and exon for all genes were quantified using the RSEM program (v1.3.3)^30^ and HTSeq framework (v2.0.2),^31^ respectively.

### Isoform Expression Analysis

The expression levels for each gene isoform were compared among the three groups of patients with FECD with TNR expansion (hereinafter defined as the “Expansion” group), patients with FECD without TNR expansion (hereinafter defined as the “No Expansion” group) and a non-FECD control group (hereinafter defined as the “Control” group) using the DESeq2 package (v1.34.0) from Bioconductor (https://www.bioconductor.org/).^32^ The DESeq2 analysis results were used to extract 93 *TCF4* isoforms listed in the Ensembl database. As the extraction criteria, isoforms with thresholds of |Log_2_ Fold Change| ≥ 1.5 and *P* value < 0.05 calculated by Wald test were considered to represent altered *TCF4* isoforms. The altered expression patterns of the *TCF4* isoforms in the three datasets were depicted using Venn diagrams using the VennDiagram package (v1.7.3) in R (v4.0.3). The expression levels of the *TCF4* isoforms that were dysregulated in common in the three datasets were visualized as boxplots using the ggplot2 package (v3.4.2) in R.

### Exon Expression Analysis

The exon usages in the *TCF4* gene region were extracted from the cleaned RNA-Seq data and subjected to DEU analysis among the three groups using the DEXSeq package (v1.46.0) from Bioconductor (https://www.bioconductor.org/).^33^ Significant DEU events were identified by setting the criteria for altered expression of *TCF4* exons as the thresholds of |Log_2_ Fold Change| ≥ 0.5 and as adjusted *P-*values < 0.05, calculated using the DEXSeq package in R. The distribution of exon expression levels was depicted as volcano plots using the ggplot2 package and the average DEU plot using the plotDEXSeq() function of the DEXSeq package in R. DEU events altered in common in the three datasets were displayed as Venn diagrams using the VennDiagram packages in R. The expression levels of DEU events common to all three datasets were visualized as boxplots using GraphPad Prism 10 software (GraphPad Software Inc., San Diego, CA, USA).

## Results

### Analysis of the Isoform-Level Differential Expression of *TCF4*

Comparison of the data between the No Expansion and Control groups using our RNA-Seq dataset (Nakagawa 2023) identified three significantly upregulated and two significantly downregulated *TCF4* isoforms (Table). Conversely, a similar comparison of the data between the Expansion and Control groups revealed that one *TCF4* isoform was significantly upregulated and one was significantly downregulated (Table).

**Table.** The Expression Level of *TCF4* Isoforms Is Significantly Altered in the Patients From the Nakagawa 2023 Dataset With FECD With or Without TNR Expansion Compared to Control Patients

Study	Expression	Ensembl ID	*TCF4* isoform^*^	Log_2_ Fold Change^†^	*P* Value^†^
No Expansion vs. Control	Upregulated	ENST00000637169	*TCF4-283*	1.53	1.93 × 10^−^^3^
		ENST00000563686	*TCF4-218*	1.67	1.90 × 10^−^^2^
		ENST00000626631	*TCF4-255*	1.54	2.10 × 10^−^^2^
	Downregulated	ENST00000635990	*TCF4-276*	−24.4	3.28 × 10^−^^16^
		ENST00000643689	*TCF4-290*	−7.13	1.04 × 10^−^^2^
Expansion vs. Control	Upregulated	ENST00000636400	*TCF4-277*	2.35	1.46 × 10^−^^2^
	Downregulated	ENST00000566286	*TCF4-232*	−7.00	4.61 × 10^−^^3^

The Nakagawa 2023 dataset is RNA-Seq data previously reported by our group.^24^^,^^25^

* Names of the isoforms are shown as a transcript name from Ensembl.

† Calculated using the Wald Test of DESeq2.

**Figure 1.**
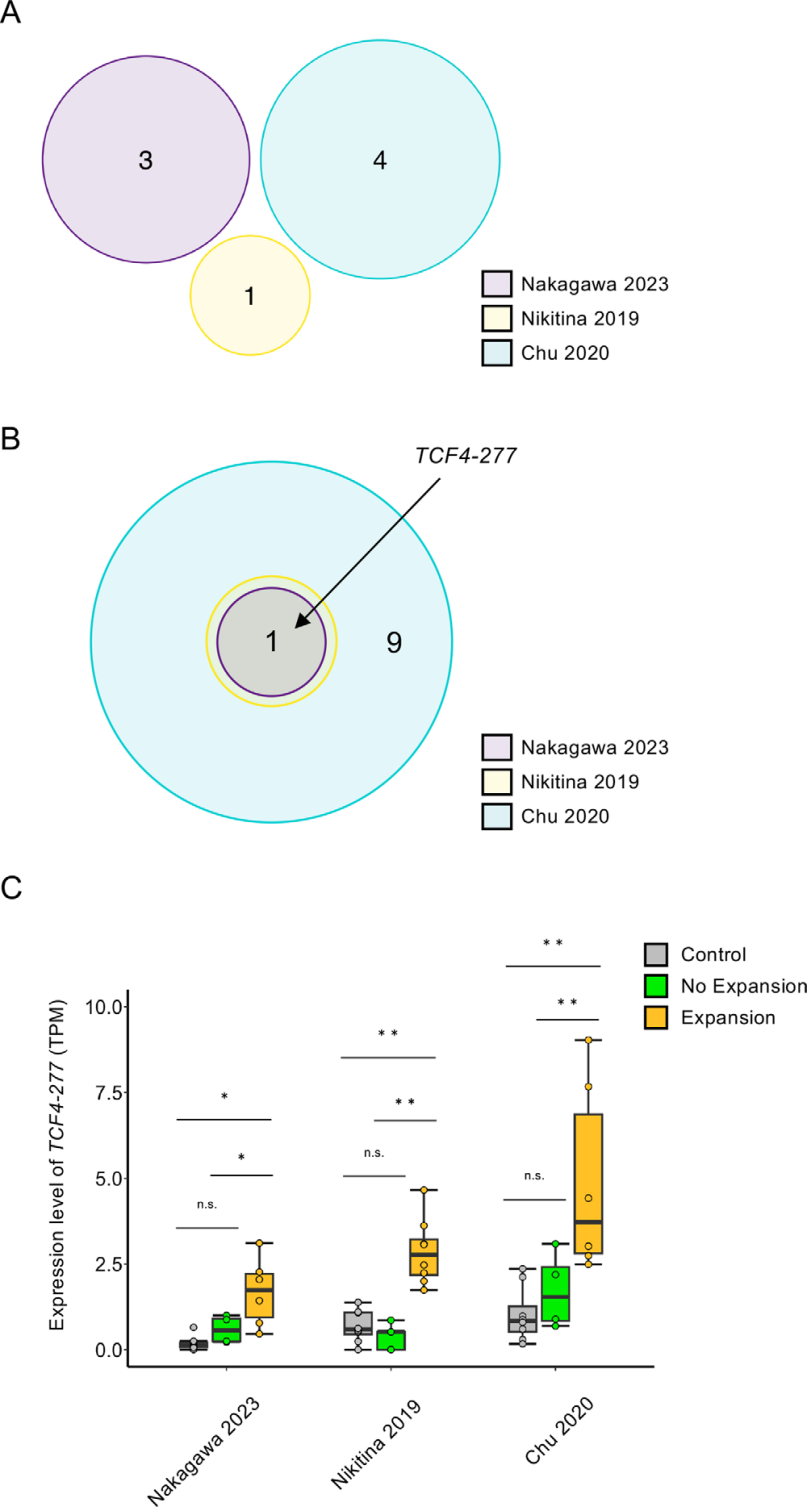
Identification of dysregulated *TCF4* isoforms in three previously reported RNA-Seq datasets. (**A**) Comparison of the No Expansion and Control groups revealed significant upregulation of the *TCF4* gene for three isoforms in the Nakagawa 2023 (*purple*), one isoform in the Nikitina 2019 (*yellow*), and four isoforms in the Chu 2020 (*blue*) datasets. However, the Venn diagram shows no upregulation of *TCF4* isoforms in common in the three datasets. (**B**) The Venn diagram shows that only one *TCF4* isoform, *TCF4-277* (ENST00000636400.2), was commonly upregulated in the Expansion compared to Control groups for all three datasets, whereas eight other upregulated isoforms were identified in Chu 2020 (*blue*). (**C**) For all three datasets, box plots show the distribution of the expression level in the *TCF4-277* isoform in the Expansion (*orange*), No Expansion (*green*), and Control (*gray*) groups (TPM = transcripts per million). Statistical analysis was performed using the Wald test in DESeq2. **P < 0.01, *P < 0.05.

We also further analyzed the differential expression of the *TCF4* isoforms using two RNA-Seq datasets (Nikitina 2019 and Chu 2020), which were previously reported by other research groups.^26^^,^^27^ The three *TCF4* isoforms found upregulated in the No Expansion compared to Control groups in the Nakagawa 2023 dataset did not show differential expression in the Nikitina 2019 and Chu 2020 datasets (Fig. 1A). Only one isoform, designated as *TCF4-277* (Ensembl ID: ENST00000636400.2), was identified as significantly upregulated in all three datasets when comparing the Expansion and Control groups (Fig. 1B). Notably, the expression level of *TCF4-277* , when comparing the Expansion and Control groups, was upregulated 2.35-, 1.73-, and 2.37-fold in the Nakagawa 2023, Nikitina 2019, and Chu 2020 datasets, respectively. By contrast, *TCF4-277* showed no significant differences when comparing the No Expansion and Control groups in any of the three datasets (Fig. 1C). None of the downregulated *TCF4* isoforms evident in the Nakagawa 2023 dataset showed differential expression in the other two RNA-Seq datasets.

### Identification of the Altered Expression of *TCF4* Exons

**Figure 2.**
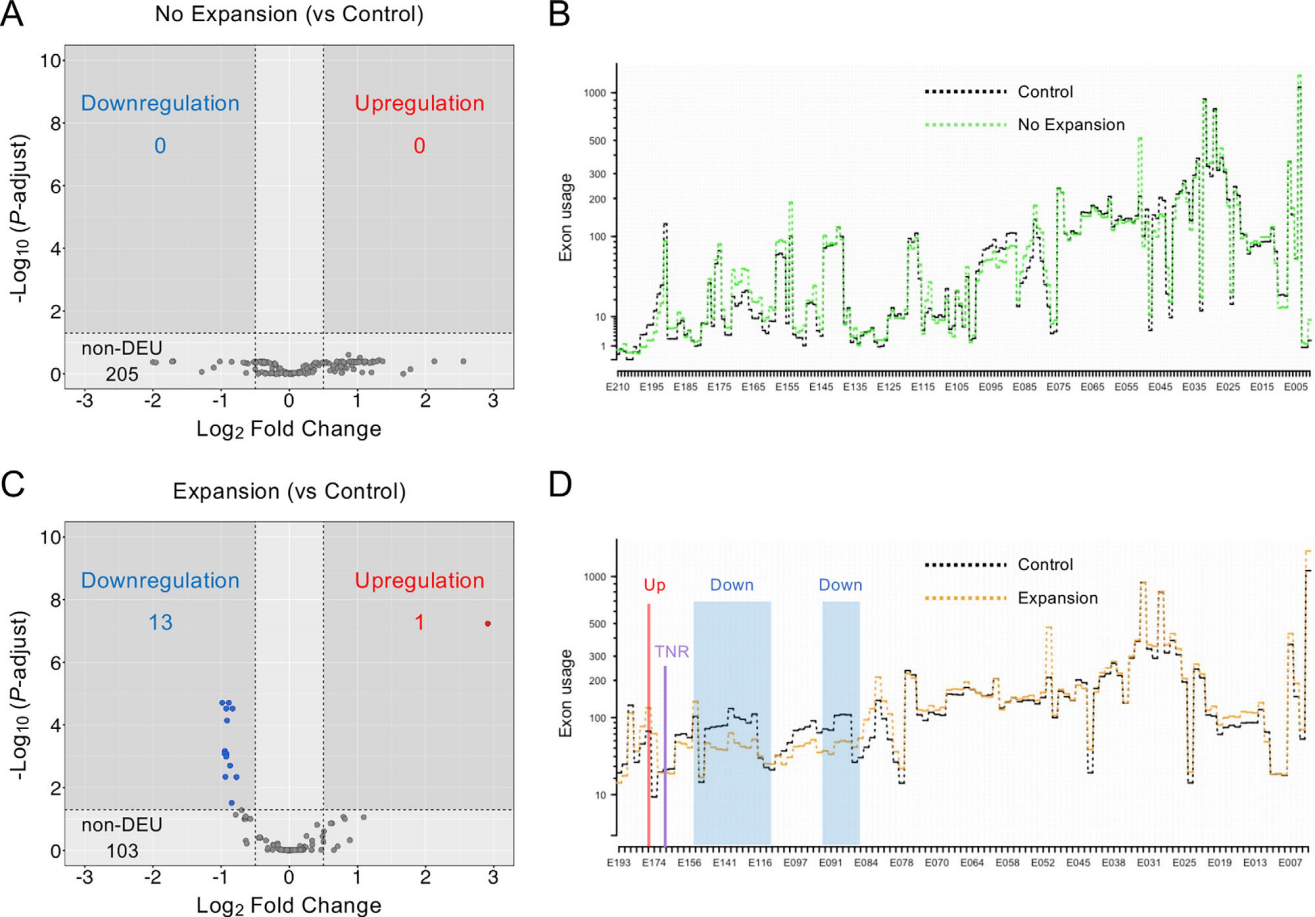
Identification of the differential exon usages (DEUs) of *TCF4* in CECs of patients with FECD. (**A**) Volcano plots show the results of DEU analysis for *TCF4* in CECs of the No Expansion group compared to the Control group in the Nakagawa 2023 dataset. Each dot indicates a DEU. The *gray-shaded areas* indicate the areas of threshold as |Log_2_ Fold Change| ≥ 0.5 and adjusted *P* values < 0.05. (**B**) Average exon usages for the No Expansion (*green dotted line*) and Control (*black dotted line*) groups show a similar pattern after plotting the DEXSeq results. (**C**) Volcano plots show 1 upregulated exon and 13 downregulated exons that differ between the Expansion and Control groups. (**D**) Average exon usages for the Expansion (*orange dotted line*) and Control (*black dotted line*) groups. Significantly upregulated or downregulated exons are highlighted with a *red line* and *blue shading*, respectively. The location of the TNR is depicted by a *purple vertical line*. Note that exons in the X-axis at **B** and **D** are numbered in reverse order because the *TCF4* gene is located in the reverse orientation compared to the reference genome.

The DEU analysis using our Nakagawa 2023 dataset did not reveal any DEUs of *TCF4* in CECs when comparing the No Expansion and Control groups, whereas a total of 205 non-DEU events were found (Fig. 2A). Consistent with the volcano plot, the exon usage patterns were similar for the No Expansion and Control groups (Fig. 2B). By contrast, one upregulated exon, E174, and 13 downregulated exons in the *TCF4* gene region were identified by comparing the Expansion and Control groups (Fig. 2C). The average exon usage data (Fig. 2D) indicated that the upregulated region (shown as the red line) in the Expansion versus the Control group was located upstream of the TNR, whereas the downregulated region (shown as blue shading) was located downstream of the TNR.

**Figure 3.**
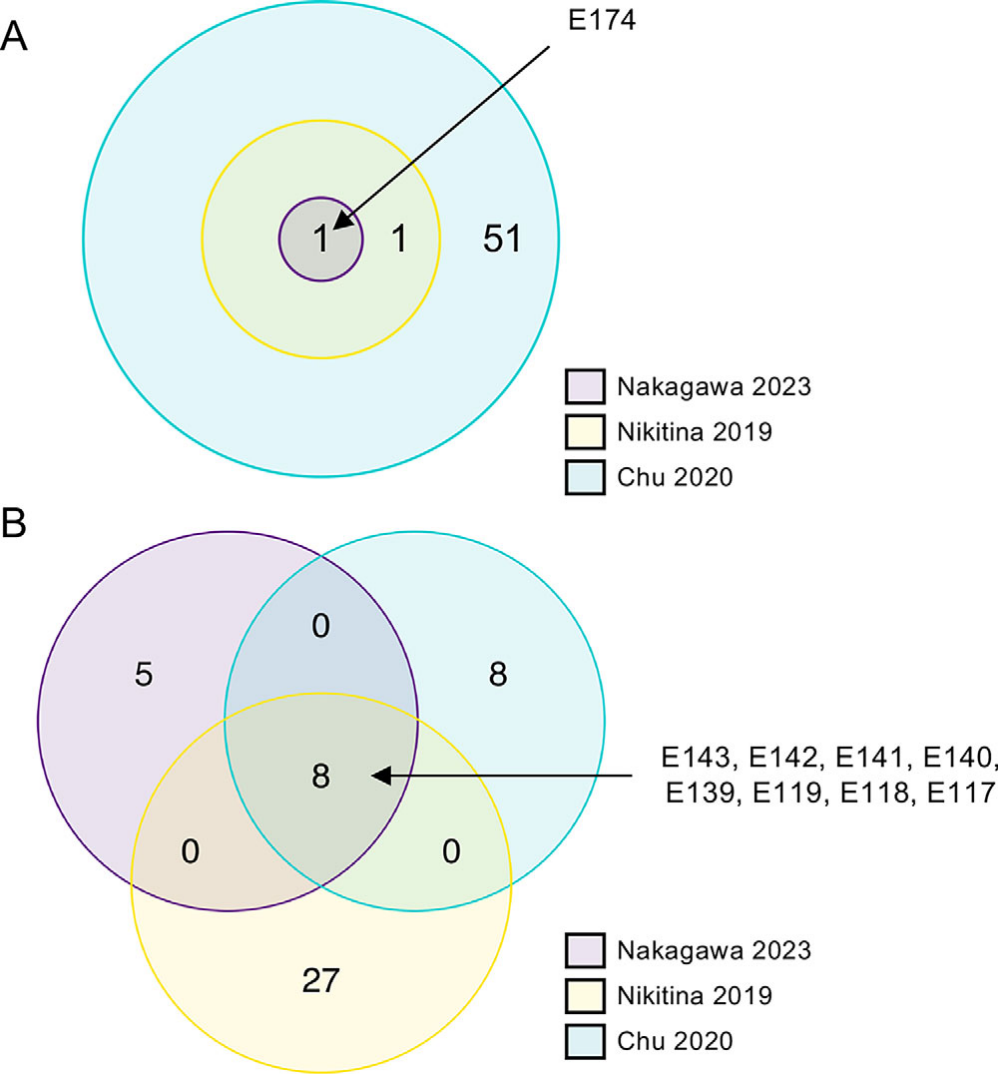
DEU results obtained by comparing the Expansion and Control groups in all three datasets. The significantly dysregulated *TCF4* exons identified by DEU analysis and comparison of the Expansion and Control groups are summarized in the Venn diagram of three circles for the Nakagawa 2023 (*purple*), Nikitina 2019 (*yellow*), and Chu 2020 (*blue*) datasets for upregulation (**A**) and downregulation (**B**). Note that only one upregulated exon (E174) was identified in all three datasets.

We also examined the DEU events in *TCF4* using the other two datasets, Nikitina 2019 and Chu 2020. The upregulated exon E174 identified in the Expansion group of Nakagawa 2023 was also found in both the Nikitina 2019 and Chu 2020 datasets (Fig. 3A). Of the 13 downregulated exons found in the Expansion group in the Nakagawa 2023 dataset, 8 exons (E143, E142, E141, E140, E139, E119, E118, and E117) were downregulated in common in the other two datasets (Fig. 3B).

**Figure 4.**
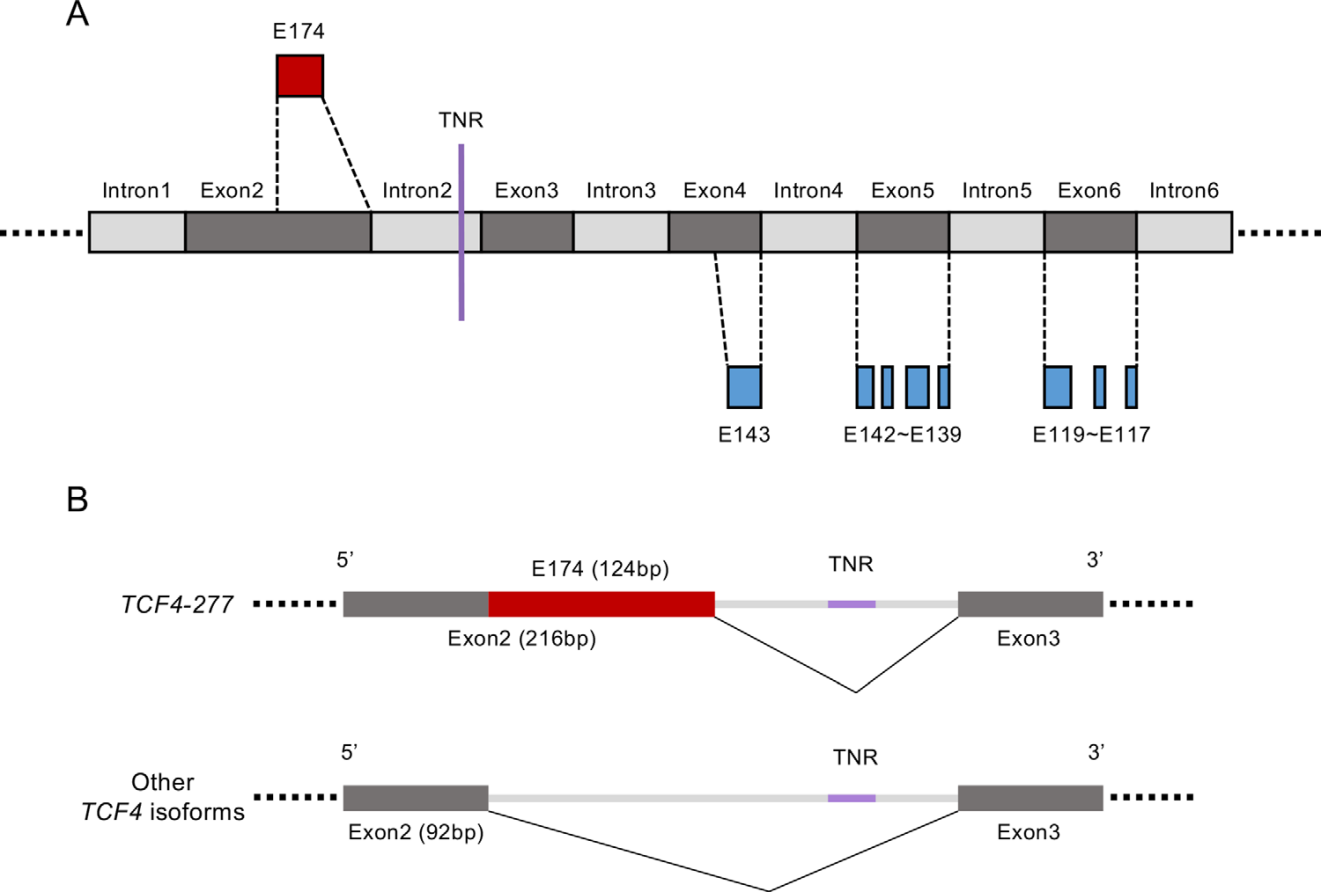
A schematic image of the *TCF4* gene with the dysregulated exons in the Expansion group. (**A**) The schematic image shows part of the *TCF4* gene from Intron1 to Intron6 based on the *TCF4-277* isoform (ENST00000636400.2). The region of the TNR, which has a previously reported association with FECD, is indicated by the *purple vertical line*. The commonly upregulated exon in all datasets is displayed in *red* above the schematic gene. Similarly, the downregulated exons are displayed in *blue* below the schematic gene. (**B**) *TCF4-277* (ENST00000636400.2), which was a significantly upregulated isoform of *TCF4* identified in all three datasets, is the only isoform that encompasses E174 located at the end of Exon2. No other isoforms encompass this exon region.

Interestingly, E174, which was upregulated in common in all three datasets, was located upstream of the TNR region (Fig. 4A). Notably, only the *TCF4-277* isoform encompassed this E174 exon; no other isoforms encompassed it (Fig. 4B). By contrast, E143, E142, E141, E140, E139, E119, E118, and E117, which were downregulated in common in the three datasets, were located downstream of the TNR expansion (Fig. 4A).

### Comparison of DEU Event Expression Levels

**Figure 5.**
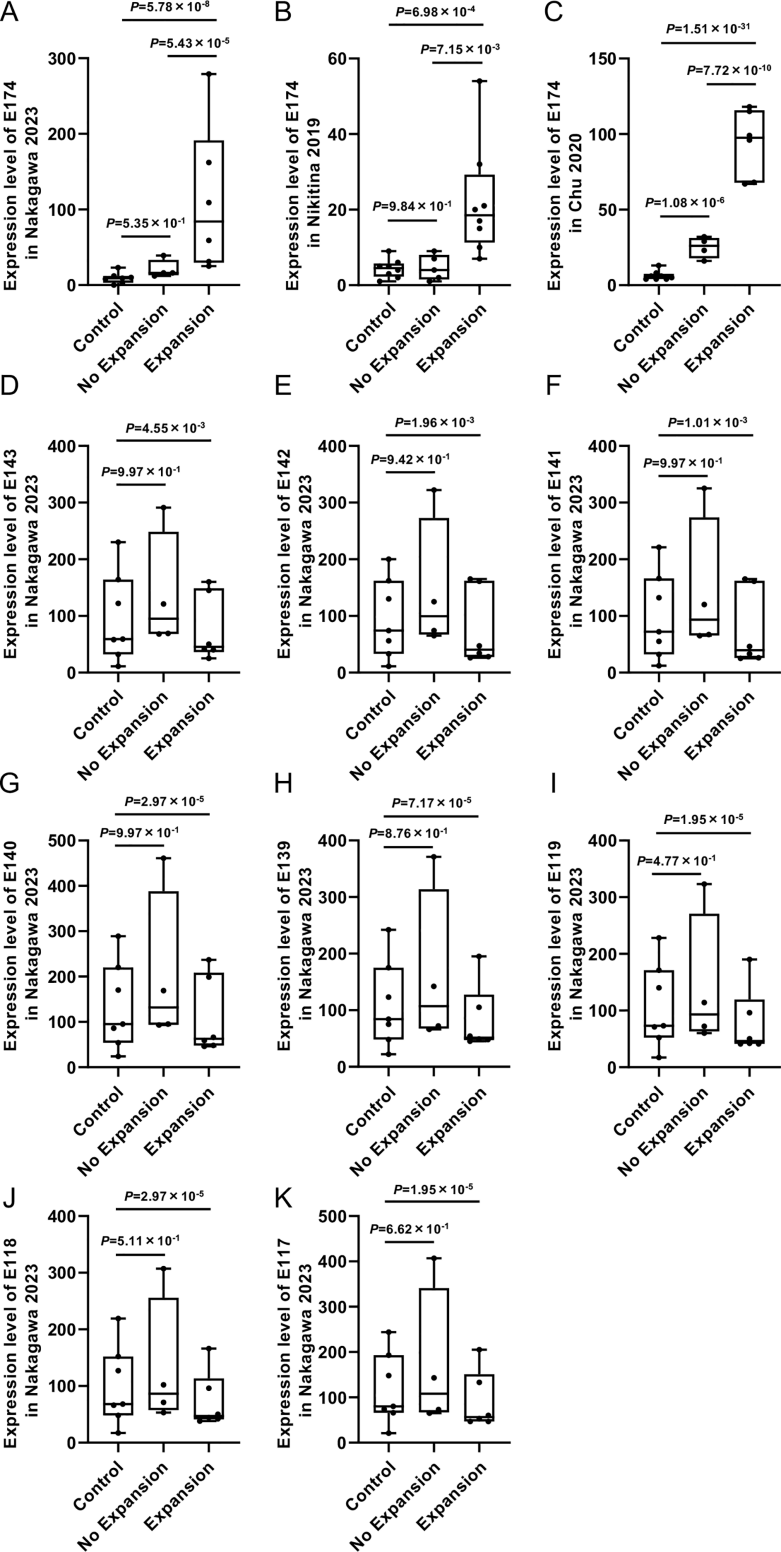
The expression level of DEU events in the *TCF4* gene in the Expansion and Control groups. Boxplot shows the expression level of upregulated E174 exon comparing the Expansion and Control groups in the Nakagawa 2023 (**A**), Nikitina 2019 (**B**), and Chu 2020 (**C**) datasets. The downregulated exons in the Nakagawa 2023 dataset are E143 (**D**), E142 (**E**), E141 (**F**), E140 (**G**), E139 (**H**), E119 (**I**), E118 (**J**), and E117 (K). The Y-axis indicates the expression level for each exon calculated using the DEXSeq package in R. Statistical analysis was also performed using the function of DEXSeq.

The expression level of E174 determined by RNA-Seq data was significantly higher in the Expansion group than in the Control group in the Nakagawa 2023 dataset (2.91-fold, Fig. 5A), in the Nikitina 2019 dataset (1.60-fold, Fig. 5B), and in the Chu 2020 dataset (3.77-fold, Fig. 5C). Conversely, E174, which showed significant upregulation in the No Expansion group, was only upregulated 1.63-fold in the Chu 2020 dataset. For the downregulated exons, E143, E142, E141, E140, E139, E119, E118, and E117 were significantly downregulated by 0.78-, 0.87-, 0.93-, 0.83-, 0.91-, 0.99-, 0.93-, and 0.89-fold in the Expansion group versus the Control group (Figs. 5D–K). The same eight exons were not significantly altered in the Expansion group in the Nikitina 2019 (Supplementary Fig. S1) and Chu 2020 (Supplementary Fig. S2) datasets.

## Discussion

Analysis of our RNA-Seq data showed that three *TCF4* isoforms were upregulated and two *TCF4* isoforms were downregulated in patients with FECD without TNR expansion compared with the control group. However, no dysregulated *TCF4* isoforms were found in common among the three RNA-Seq datasets, which included ours. We found that one *TCF4* isoform was upregulated and one *TCF4* isoform was downregulated when we compared the RNA-Seq data from the patients with FECD with the TNR expansion and the control group. Notably, further analysis using all three datasets identified that one isoform, namely *TCF4-277* (ENST00000636400.2), was upregulated in common. In addition, we showed that one exon (E174) located upstream of TNR, which encompassed *TCF4-277*, was upregulated in common in all three datasets, whereas eight exons located downstream of the TNR were downregulated in common.

We previously hypothesized that the *TCF4* transcript is affected by the presence of the TNR expansion (which presumably promotes downregulation by interfering with transcription). We therefore assessed the transcript level of *TCF4* using three qPCR probes that are all contained within the canonical *TCF4* transcript that encodes *TCF4-B* (ENST00000354452.8). Our qPCR data, derived from 35 controls, 41 patients with FECD without the TNR expansion, and 162 patients with FECD with the TNR expansion, demonstrated that *TCF4* expression is significantly higher in the FECD with TNR expansion group than in the FECD without TNR expansion group or the control group, which refuted our original hypothesis.^17^ However, Oldak and colleagues^18^ found no significant differences in *TCF4* expression levels between 40 patients with FECD and 23 controls. In their qPCR analysis, they used a probe for the coding region, which is present in both *TCF4-A* (ENST00000457482.7) and *TCF4-B* (ENST00000354452.8), located in the amino-terminal part of the protein close to the activation domain 2.^18^ Mootha and colleagues^19^ also reported no significant differences in *TCF4* expression between five patients with FECD with TNR and five controls, using primers specific to the constitutively expressed exon encoding the bHLH domain present in all *TCF4* protein isoforms. Conversely, Foja and colleagues^20^ used a single TaqMan probe complementary to an exon region close to the TNR region and found downregulation of *TCF4* when comparing five patients with FECD with TNR expansion and six controls. One potential explanation for these inconsistencies in the expression level of *TCF4* transcripts is the existence of 93 isoforms with variable expression levels, because qPCR has the limitation of assessing the expression levels of high numbers of isoforms that share the same sequences to some extent.

Sirp and colleagues^22^ previously reported two RNA-Seq data and showed downregulation of *TCF4* isoforms transcribed from the alternative 5′ exons in the proximity of the TNR, but upregulation of other *TCF4* isoforms in patients with FECD with TNR expansion*.* Likewise, long-read RNA-Seq showed lower expression of *TCF4* isoforms located downstream of TNR in three patients with FECD with TNR expansion than in three controls.^34^ The digital droplet PCR also revealed that patients with FECD had a lower percentage of the ratio of the *TCF4* transcript spanning over the TNR to the total *TCF4* gene expression.^23^ Consistently, our current analysis demonstrated that dysregulation of *TCF4* in the patients with TNR expansion varies depending on the isoforms. Taken together, these accumulating data support the concept that the expression levels of *TCF4* isoforms are affected by the presence of the TNR expansion.

Because exon usage in RNA splicing plays a crucial role in generating isoforms, the effect of TNR repeats on exon usage has also been investigated. For instance, an RNA-Seq investigation of a coverage plot of the average number of RNA-Seq reads in *TCF4* genes showed that sequences located upstream of TNR preferentially accumulated in the patients with FECD with the TNR expansion compared to the patients with FECD without TNR expansion or a control group.^35^ As also shown by qPCR, the intronic RNA upstream of TNR was upregulated in the CECs of the six patients with TNR expansions when compared to 10 controls.^36^ In the current study, our comparison of the Expansion group and the control identified one upregulated exon (E174) located upstream of the TNR and 8 exons (E143, E142, E141, E140, E139, E119, E118, and E117) located downstream of the TNR that were downregulated. Our data show that no exon usage on *TCF4* was significantly altered in the Expansion group compared to the control group, indicating that TNR induces dysregulated exon usage. The finding that E174 is only encompassed by *TCF4-277*, but not other isoforms, was also consistent with our finding that *TCF4-277* was the only upregulated isoform in the Expansion group in all three RNA-Seq datasets analyzed in this study.

Isoforms that result in proteome diversity are generated by alternative splicing as the key mechanism and occur in almost all multiexon genes in humans.^37^ Regulated production of splice variants is a crucial contributor to various biological processes; therefore, mutations affecting sequences that are involved in splicing can induce diseases.^38^^–^^43^ Indeed, splicing dysregulation is a key factor in the development of many diseases, such as cancer, cardiomyopathy, cardiac hypertrophy, autism spectrum disorder, spinal muscular atrophy, schizophrenia, Duchenne muscular dystrophy, liver disease, and chronic kidney disease.^38^^–^^45^ In addition, therapeutic strategies targeting splicing dysregulation, including protocols for restoring open reading frames, influencing alternative splicing, and inducing exon inclusion, have been investigated.^44^^,^^46^^–^^48^ Consequently, multiple small-molecule splicing modulators have been developed especially for use as novel cancer therapies. No approved drug is currently approved; however, the safety and efficacy of pre-mRNA splicing modulators, including SF3b inhibitor (NCT02841540), SRPK inhibitor (NCT04247256), CLK inhibitor (NCT03355066 and NCT05732103), CDK inhibitor (NCT01580228, NCT04555473, and others), and PRMT5 inhibitor (NCT5094336, NCT03614728, and others), have been evaluated in clinical trials.^48^ Further investigations aimed at elucidating the pathophysiological effect of pre-mRNA splicing dysregulation on FECD will be interesting and may identify potential therapeutic targets.

The limitation of the present study is that our analysis using short-read RNA-Seq data evaluated only the 93 known *TCF4* isoforms, leaving open the possibility that other novel and as-yet-unidentified pathological isoforms may also have roles in FECD. Future investigation utilizing long-read RNA-Seq may provide a clearer picture of the splicing landscape, as longer reads are particularly advantageous for identifying and characterizing novel splicing events.^49^^–^^51^ Moreover, this study did not clarify the target genes and pathways dysregulated by the *TCF4-277* isoform. Although gene ontology and KEGG pathway analyses typically provide substantial insights, their databases lack the granularity to distinguish isoform-specific impacts. Consequently, further in vitro and in vivo research is necessary to determine whether *TCF4-277* is implicated in the pathological phenotype of FECD and to establish its potential causative role. In summary, we succeeded in identifying one dysregulated isoform of *TCF4* in FECD with TNR expansion. Further studies are anticipated, because dysregulated splicing events in *TCF4,* resulting in the upregulation of a specific isoform located upstream of the TNR, could represent the potential cause of FECD and are worth researching as potential therapeutic targets.

## Supplementary Material

Supplement 1

Supplement 2

Supplement 3

Supplement 4

Supplement 5
