# Supplementary material for: Dysregulation of the TCF4 Isoform in Corneal Endothelial Cells of Patients With Fuchs Endothelial Corneal Dystrophy
Source: Invest Ophthalmol Vis Sci. 2024 Jun 17;65(6):27. doi: 10.1167/iovs.65.6.27 (PMC11185267; doi:10.1167/iovs.65.6.27)
Supplement: Supplement 2 [file iovs-65-6-27_s002.pdf]

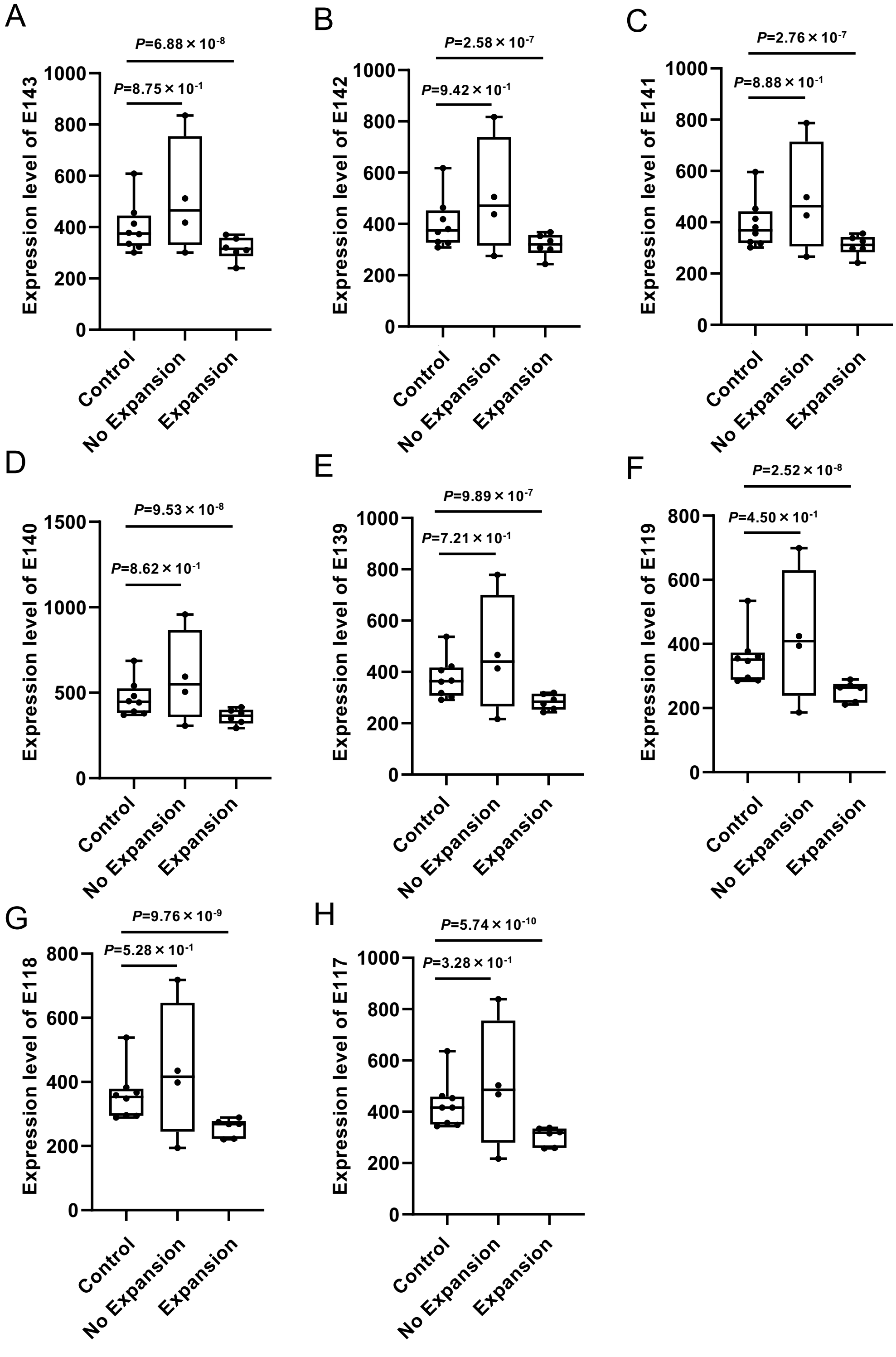

Supplemental Figure 2. The expression levels of differential exon usage (DEU) events in the *TCF4* gene in the RNA-Seq data of Chu 2020

Expression levels of E143, E142, E141, E140, E139, E119, E118, and E117, which were commonly identified in three RNA-Seq datasets as downregulated exons in Fuchs endothelial corneal dystrophy (FECD) with expansion. (A) E143, (B) E142, (C) E141, (D) E140, (E) E139, (F) E119, (G) E118, and (H) E117 showed 0.52-, 0.52-, 0.53-, 0.56-, 0.59-, 0.67-, 0.66-, and 0.68-fold downregulation, respectively, in the FECD with expansion group compared to the control group. The same 8 exons were not significantly altered in the FECD without expansion group. The RNA-Seq data analyzed in this supplemental figure were previously reported by Chu and colleagues.[27]
