# Supplementary material for: Dysregulation of the TCF4 Isoform in Corneal Endothelial Cells of Patients With Fuchs Endothelial Corneal Dystrophy
Source: Invest Ophthalmol Vis Sci. 2024 Jun 17;65(6):27. doi: 10.1167/iovs.65.6.27 (PMC11185267; doi:10.1167/iovs.65.6.27)
Supplement: Supplement 3 [file iovs-65-6-27_s003.pdf]

Supplemental Table 1. Sample information of RNA-Seq data from Nakagawa 2023

Group	Sample ID	Age	Sex
Control	S1	69	Female
Control	S6	62	Female
Control	S8	69	Male
Control	S16	57	Female
Control	S20	48	Male
Control	S23	64	Female
Control	S28	59	Male
No Expansion	FECD662	77	Male
No Expansion	FECD681	63	Female
No Expansion	FECD693	78	Male
No Expansion	FECD697	61	Female
Expansion	FECD665	67	Male
Expansion	FECD666	79	Female
Expansion	FECD687	53	Male
Expansion	FECD690	64	Male
Expansion	FECD691	68	Female
Expansion	FECD699	64	Female

No Expansion: CTG expansion < 50
Expansion: CTG expansion ≥ 50
