# Supplementary material for: Dysregulation of the TCF4 Isoform in Corneal Endothelial Cells of Patients With Fuchs Endothelial Corneal Dystrophy
Source: Invest Ophthalmol Vis Sci. 2024 Jun 17;65(6):27. doi: 10.1167/iovs.65.6.27 (PMC11185267; doi:10.1167/iovs.65.6.27)
Supplement: Supplement 4 [file iovs-65-6-27_s004.pdf]

Supplemental Table 2. Sample information of RNA-Seq data from Nikitina 2019

| Group | Sample ID | Age | Sex |
|---|---|---|---|
| Control | C_201 | 47 | Female |
| Control | C_202 | 54 | Male |
| Control | C_203 | 63 | Male |
| Control | C_204 | 59 | Male |
| Control | C_205 | 54 | Female |
| Control | C_206 | 65 | Female |
| Control | C_207 | 64 | Female |
| Control | C_208 | 61 | Male |
| No Expansion | Dfu_209 | 56 | Female |
| No Expansion | Dfu_210 | 64 | Male |
| No Expansion | Dfu_217 | 54 | Female |
| No Expansion | Dfu_219 | 69 | Female |
| No Expansion | Dfu_223 | 79 | Male |
| Expansion | Dfu_201 | 70 | Female |
| Expansion | Dfu_202 | 63 | Male |
| Expansion | Dfu_203 | 57 | Female |
| Expansion | Dfu_205 | 64 | Female |
| Expansion | Dfu_207 | 79 | Female |
| Expansion | *Dfu_212 | 72 | Female |
| Expansion | Dfu_213 | 74 | Male |
| Expansion | Dfu_215 | 70 | Female |

No Expansion: CTG expansion < 50
Expansion: CTG expansion ≥ 50
* Dfu_212 harbors 44 times of CTG repeat expansion
