# Supplementary material for: Dysregulation of the TCF4 Isoform in Corneal Endothelial Cells of Patients With Fuchs Endothelial Corneal Dystrophy
Source: Invest Ophthalmol Vis Sci. 2024 Jun 17;65(6):27. doi: 10.1167/iovs.65.6.27 (PMC11185267; doi:10.1167/iovs.65.6.27)
Supplement: Supplement 5 [file iovs-65-6-27_s005.pdf]

Supplemental Table 3. Sample information of RNA-Seq data from Chu 2020

Group	Sample ID	Age	Sex
Control	Control_0	74	Female
Control	Control_1	72	Female
Control	Control_2	51	Male
Control	Control_3	68	Male
Control	Control_4	68	Male
Control	Control_5	71	Male
Control	Control_6	61	Female
Control	Control_7	74	Male
No Expansion	FECD_NR_0	71	Female
No Expansion	FECD_NR_1	72	Female
No Expansion	FECD_NR_2	51	Female
No Expansion	FECD_NR_3	71	Female
Expansion	FECD_REP_0	67	Female
Expansion	FECD_REP_1	66	Male
Expansion	FECD_REP_2	68	Female
Expansion	FECD_REP_3	58	Female
Expansion	FECD_REP_4	69	Female
Expansion	FECD_REP_5	71	Female

No Expansion: CTG expansion < 50
Expansion: CTG expansion ≥ 50
